# 4′-Phenyl-3,4-di­hydro-2*H*-spiro­[naph­tha­lene-1,3′-[1,2,4]triazole]-5′-thione

**DOI:** 10.1107/S1600536814012409

**Published:** 2014-05-31

**Authors:** Joel T. Mague, Mehmet Akkurt, Shaaban K. Mohamed, Alaa A. Hassan, Mustafa R. Albayati

**Affiliations:** a Department of Chemistry, Tulane University, New Orleans, LA 70118, USA; b Department of Physics, Faculty of Sciences, Erciyes University, 38039 Kayseri, Turkey; c Chemistry and Environmental Division, Manchester Metropolitan University, Manchester M1 5GD, England; d Chemistry Department, Faculty of Science, Minia University, 61519 El-Minia, Egypt; e Chemistry Department, Faculty of Science, Mini University, 61519 El-Minia, Egypt; f Kirkuk University, College of Science, Department of Chemistry, Kirkuk, Iraq

## Abstract

In the title mol­ecule, C_17_H_15_N_3_S, the phenyl group makes a dihedral angle of 57.29 (11)° with the mean plane of the triazole ring, which in turn makes an angle of 86.83 (12)° with the plane of the aromatic portion of the tetra­hydro­naphthalene moiety. In the crystal, mol­ecules are linked by weak C—H⋯S hydrogen bonds into supra­molecular chains propagating along the *a*-axis direction. Weak C—H⋯π inter­actions are also observed.

## Related literature   

For the synthesis of different triazole thione compounds, see: Wujec *et al.* (2004 ▶); Zamani *et al.* (2004 ▶); Pitucha *et al.* (2007 ▶); Farghaly & El-Kashef (2006 ▶); Guelerman *et al.* (1998 ▶); Salgin-Gökşen *et al.* (2007 ▶). For the biological activity of triazole thio­nes, see: Amir & Kumar (2007 ▶); Gokce *et al.* (2001 ▶); Ezabadi *et al.* (2008 ▶); Mazzone *et al.* (1981 ▶); Küçükgüzel *et al.* (2008 ▶); Dogan *et al.* (2005 ▶); Kane *et al.* (1994 ▶); Kane *et al.* (1988 ▶). For ring-puckering parameters, see: Cremer & Pople (1975 ▶).
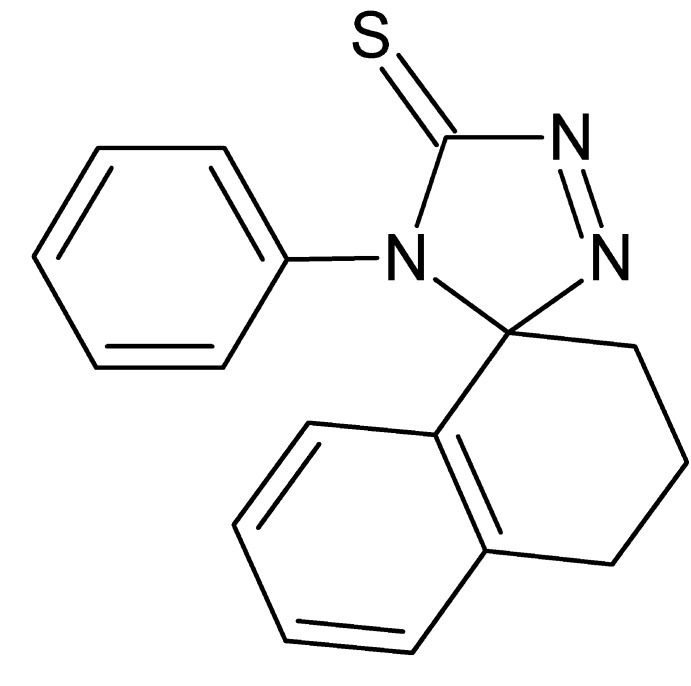


## Experimental   

### 

#### Crystal data  

C_17_H_15_N_3_S
*M* *_r_* = 293.39
Orthorhombic, 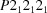

*a* = 6.2091 (9) Å
*b* = 13.1804 (19) Å
*c* = 17.391 (3) Å
*V* = 1423.3 (4) Å^3^
*Z* = 4
Mo *K*α radiation
μ = 0.22 mm^−1^
*T* = 150 K
0.25 × 0.15 × 0.08 mm

#### Data collection  

Bruker SMART APEX CCD diffractometer
Absorption correction: multi-scan (*SADABS*; Bruker, 2013 ▶) *T*_min_ = 0.80, *T*_max_ = 0.98
25736 measured reflections
3564 independent reflections
3274 reflections with *I* > 2σ(*I*)
*R*_int_ = 0.048

#### Refinement  

*R*[*F* ^2^ > 2σ(*F* ^2^)] = 0.035
*wR*(*F* ^2^) = 0.085
*S* = 1.04
3564 reflections
190 parameters
H-atom parameters constrained
Δρ_max_ = 0.26 e Å^−3^
Δρ_min_ = −0.15 e Å^−3^
Absolute structure: Flack *x* determined using 1312 quotients [(*I* ^+^)−(*I* ^−^)]/[(*I* ^+^)+(*I* ^−^)] (Parsons *et al.*, 2013 ▶)
Absolute structure parameter: −0.02 (3)

### 

Data collection: *APEX2* (Bruker, 2013 ▶); cell refinement: *SAINT* (Bruker, 2013 ▶); data reduction: *SAINT*; program(s) used to solve structure: *SHELXTL* (Sheldrick, 2008 ▶); program(s) used to refine structure: *SHELXTL*; molecular graphics: *ORTEP-3 for Windows* (Farrugia, 2012 ▶); software used to prepare material for publication: *WinGX* (Farrugia, 2012 ▶) and *PLATON* (Spek, 2009 ▶).

## Supplementary Material

Crystal structure: contains datablock(s) global, I. DOI: 10.1107/S1600536814012409/xu5795sup1.cif

Structure factors: contains datablock(s) I. DOI: 10.1107/S1600536814012409/xu5795Isup2.hkl

Supporting information file. DOI: 10.1107/S1600536814012409/xu5795Isup3.cml

CCDC reference: 1005633

Additional supporting information:  crystallographic information; 3D view; checkCIF report

## Figures and Tables

**Table 1:** Hydrogen-bond geometry (Å, °) *Cg*3 is the centroid of the benzene ring of the 1,2,3,4-tetra­hydro­naphthalene group.

*D*—H⋯*A*	*D*—H	H⋯*A*	*D*⋯*A*	*D*—H⋯*A*
C7—H7⋯S1^i^	0.95	2.86	3.568 (3)	132
C6—H6⋯*Cg*3^ii^	0.95	2.84	3.602 (2)	138
C16—H16⋯*Cg*3^iii^	0.95	2.90	3.676 (3)	139

Symmetry codes: (i) 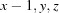; (ii) 
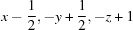
; (iii) 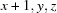.

**Fig. 1.**
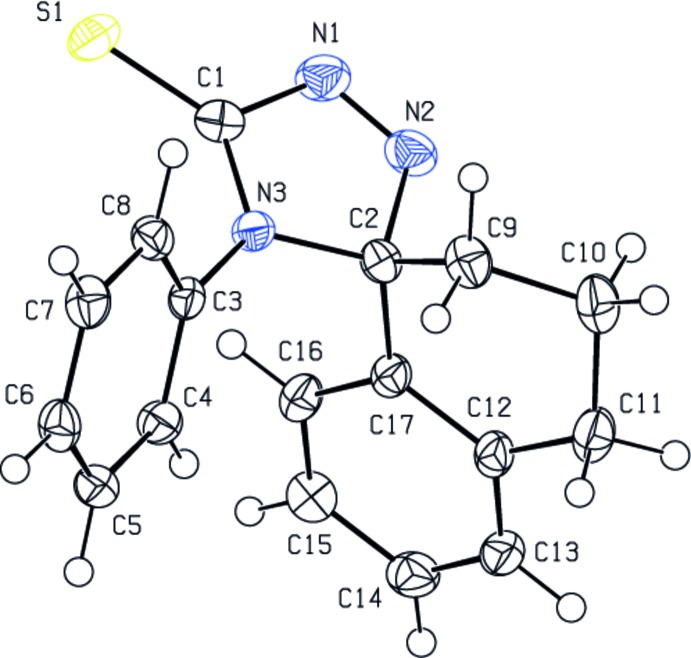
Perspective view of the title compound with 50% probability ellipsoids.

**Fig. 2.**
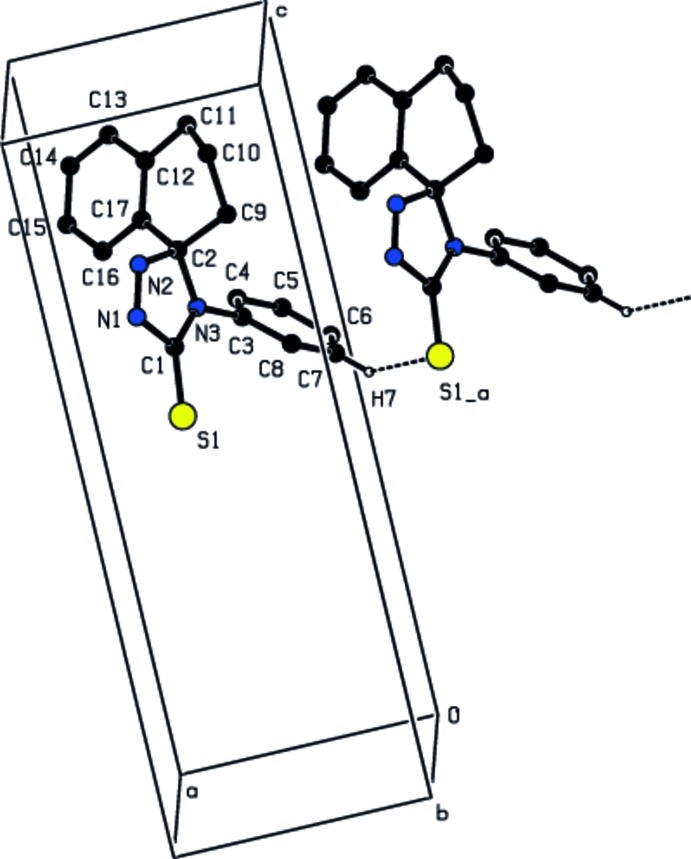
Packing viewed down the a axis. Hydrogen atoms are omitted.

## supplementary crystallographic information

### 1. Comment

Triazole thiones have been prepared by different methods based mostly on cyclodehydration of thiosemicarbazides with a variety of basic reagents such as sodium hydroxide (Wujec *et al.*, 2004; Zamani *et al.*, 2004; Pitucha *et al.*, 2007), potassium hydroxide (Farghaly & El-Kashef, 2006), sodium carbonate (Guelerman *et al.*, 1998) and triethylamine (Salgin-Gökşen *et al.*, 2007). On other hand the pharmacological properties such as anti-inflammatory, analgesic (Amir & Kumar, 2007; Gokce *et al.*, 2001), anti-bacterial, anti-fungal (Ezabadi *et al.*, 2008; Mazzone *et al.*, 1981), anti-tubercular, anti-viral (Küçükgüzel *et al.*, 2008), anti-tumoral (Dogan *et al.*, 2005), anti-convulsant (Kane *et al.*, 1994) and anti-depressant (Kane *et al.*, 1988) activities have been reported for mercapto-and thione-substituted 1,2,4-triazole systems. Based on above findings, we herein report the use of chloranil as a dehydrogenating agent of (1E)-3,4,4a,5,8,8a-hexahydronaphthalen-1(2*H*)-one *N*-phenylthiosemicarbazone to give the corresponding triazole thione compound.

In the title compound, the phenyl group (C3–C8) attached to N3 makes a dihedral angle of 57.29 (11)° with the mean plane of the triazole ring (N1–N3/C1/C2) which in turn makes an angle of 86.83 (12)° with the plane of the aromatic portion (C12–C17) of the tetrahydronaphthalene moiety (Fig. 1). A Cremer-Pople analysis of the conformation of the ring C2/C9–C12/C17 gave puckering parameters Q(2) = 0.356 (3) Å, Q(3) = -0.332 (3) Å and φ(2) = 275.4 (4)° (Cremer & Pople, 1975). In the solid there are no unusual intermolecular contacts. Only weak C—H···S and C—H···π interactions are observed (Table 1, Fig. 2).

### 2. Experimental

A mixture of 1 mmol (299 mg) of (1E)-3,4,4a,5,8,8a-hexahydronaphthalen-1(2*H*)-one *N*-phenylthiosemicarbazone and 1 mmol (246 mg) of 2,3,5,6-tetrachloro-1,4-benzoquinone in 30 ml dry ethyl acetate was stirred for 96 h at room temperature. The precipitate was filtered off, dried under vacuum and recrytallized from ethanol to give pure orange crystals.

### 3. Refinement

H atoms were placed in calculated positions with C—H = 0.95-0.99 Å, and refined in riding mode with U_iso_(H) = 1.2U_eq_(C).

### Crystal data

**Table:**

C_17_H_15_N_3_S	*F*(000) = 616
*M**_r_* = 293.39	*D*_x_ = 1.369 Mg m^−^^3^
Orthorhombic, *P*2_1_2_1_2_1_	Mo *K*α radiation, λ = 0.71073 Å
Hall symbol: P 2ac 2ab	Cell parameters from 9998 reflections
*a* = 6.2091 (9) Å	θ = 2.3–28.3°
*b* = 13.1804 (19) Å	µ = 0.22 mm^−^^1^
*c* = 17.391 (3) Å	*T* = 150 K
*V* = 1423.3 (4) Å^3^	Column, orange
*Z* = 4	0.25 × 0.15 × 0.08 mm

### Data collection

**Table:**

Bruker SMART APEX CCD diffractometer	3564 independent reflections
Radiation source: fine-focus sealed tube	3274 reflections with *I* > 2σ(*I*)
Graphite monochromator	*R*_int_ = 0.048
Detector resolution: 8.3660 pixels mm^-1^	θ_max_ = 28.3°, θ_min_ = 1.9°
φ and ω scans	*h* = −8→8
Absorption correction: multi-scan (*SADABS*; Bruker, 2013)	*k* = −17→17
*T*_min_ = 0.80, *T*_max_ = 0.98	*l* = −23→23
25736 measured reflections	

### Refinement

**Table:**

Refinement on *F*^2^	Hydrogen site location: inferred from neighbouring sites
Least-squares matrix: full	H-atom parameters constrained
*R*[*F*^2^ > 2σ(*F*^2^)] = 0.035	*w* = 1/[σ^2^(*F*_o_^2^) + (0.0378*P*)^2^ + 0.4193*P*] where *P* = (*F*_o_^2^ + 2*F*_c_^2^)/3
*wR*(*F*^2^) = 0.085	(Δ/σ)_max_ = 0.001
*S* = 1.04	Δρ_max_ = 0.26 e Å^−^^3^
3564 reflections	Δρ_min_ = −0.15 e Å^−^^3^
190 parameters	Absolute structure: Flack *x* determined using 1312 quotients [(*I*^+^)-(*I*^-^)]/[(*I*^+^)+(*I*^-^)] (Parsons *et al.*, 2013)
0 restraints	Absolute structure parameter: −0.02 (3)

### Special details

**Table:**

Geometry. Bond distances, angles *etc*. have been calculated using the rounded fractional coordinates. All su's are estimated from the variances of the (full) variance-covariance matrix. The cell e.s.d.'s are taken into account in the estimation of distances, angles and torsion angles
Refinement. Refinement on *F*^2^ for ALL reflections except those flagged by the user for potential systematic errors. Weighted *R*-factors *wR* and all goodnesses of fit *S* are based on *F*^2^, conventional *R*-factors *R* are based on *F*, with *F* set to zero for negative *F*^2^. The observed criterion of *F*^2^ > σ(*F*^2^) is used only for calculating -*R*-factor-obs *etc*. and is not relevant to the choice of reflections for refinement. *R*-factors based on *F*^2^ are statistically about twice as large as those based on *F*, and *R*-factors based on ALL data will be even larger.

### Fractional atomic coordinates and isotropic or equivalent isotropic displacement parameters (Å^2^)

**Table:**

	*x*	*y*	*z*	*U*_iso_*/*U*_eq_	
S1	0.62076 (10)	0.54064 (5)	0.53367 (3)	0.0310 (2)	
N1	0.6979 (4)	0.57824 (16)	0.68377 (12)	0.0330 (6)	
N2	0.6462 (3)	0.54877 (15)	0.74954 (11)	0.0306 (6)	
N3	0.4806 (3)	0.44434 (13)	0.66343 (10)	0.0222 (5)	
C1	0.5921 (3)	0.51596 (16)	0.62549 (13)	0.0252 (6)	
C2	0.5003 (3)	0.45985 (17)	0.74710 (12)	0.0231 (6)	
C3	0.3302 (3)	0.37544 (16)	0.62918 (11)	0.0207 (6)	
C4	0.3432 (3)	0.27253 (16)	0.64403 (12)	0.0240 (6)	
C5	0.1925 (4)	0.20741 (17)	0.61087 (13)	0.0258 (6)	
C6	0.0339 (4)	0.24503 (18)	0.56279 (12)	0.0266 (6)	
C7	0.0221 (4)	0.34798 (18)	0.54906 (12)	0.0270 (6)	
C8	0.1688 (3)	0.41375 (17)	0.58248 (12)	0.0241 (6)	
C9	0.2844 (4)	0.48971 (19)	0.78250 (13)	0.0291 (7)	
C10	0.3014 (4)	0.4969 (2)	0.86981 (14)	0.0346 (8)	
C11	0.3616 (4)	0.39487 (19)	0.90331 (13)	0.0315 (7)	
C12	0.5517 (4)	0.34643 (17)	0.86358 (12)	0.0243 (6)	
C13	0.6703 (4)	0.27160 (18)	0.90093 (13)	0.0292 (7)	
C14	0.8448 (4)	0.22515 (18)	0.86675 (14)	0.0314 (7)	
C15	0.9049 (4)	0.25245 (18)	0.79299 (14)	0.0295 (7)	
C16	0.7902 (4)	0.32656 (18)	0.75447 (12)	0.0245 (6)	
C17	0.6145 (4)	0.37395 (16)	0.78893 (11)	0.0216 (5)	
H4	0.45350	0.24650	0.67640	0.0290*	
H5	0.19880	0.13670	0.62140	0.0310*	
H6	−0.06640	0.20020	0.53940	0.0320*	
H7	−0.08750	0.37400	0.51640	0.0320*	
H8	0.15880	0.48470	0.57340	0.0290*	
H9A	0.23820	0.55600	0.76150	0.0350*	
H9B	0.17420	0.43860	0.76860	0.0350*	
H10A	0.41200	0.54780	0.88390	0.0420*	
H10B	0.16180	0.51930	0.89140	0.0420*	
H11A	0.39580	0.40350	0.95850	0.0380*	
H11B	0.23620	0.34880	0.89940	0.0380*	
H13	0.62990	0.25190	0.95150	0.0350*	
H14	0.92350	0.17460	0.89380	0.0380*	
H15	1.02430	0.22040	0.76900	0.0350*	
H16	0.83170	0.34540	0.70390	0.0290*	

### Atomic displacement parameters (Å^2^)

**Table:**

	*U*^11^	*U*^22^	*U*^33^	*U*^12^	*U*^13^	*U*^23^
S1	0.0328 (3)	0.0327 (3)	0.0274 (3)	0.0027 (3)	0.0043 (2)	0.0093 (2)
N1	0.0365 (11)	0.0290 (10)	0.0336 (11)	−0.0093 (9)	−0.0021 (9)	0.0033 (8)
N2	0.0339 (10)	0.0242 (10)	0.0336 (10)	−0.0065 (9)	−0.0046 (9)	−0.0017 (8)
N3	0.0257 (9)	0.0210 (10)	0.0199 (8)	−0.0036 (7)	−0.0028 (7)	0.0013 (7)
C1	0.0243 (10)	0.0216 (11)	0.0298 (11)	0.0001 (8)	−0.0004 (9)	0.0021 (8)
C2	0.0261 (9)	0.0217 (10)	0.0214 (10)	−0.0032 (9)	−0.0022 (8)	−0.0029 (8)
C3	0.0248 (10)	0.0223 (10)	0.0151 (9)	−0.0019 (8)	−0.0002 (8)	−0.0011 (8)
C4	0.0264 (10)	0.0238 (10)	0.0217 (9)	0.0010 (8)	−0.0047 (9)	0.0016 (8)
C5	0.0314 (11)	0.0214 (11)	0.0246 (10)	−0.0027 (9)	−0.0011 (9)	−0.0009 (8)
C6	0.0263 (10)	0.0311 (12)	0.0224 (10)	−0.0040 (9)	−0.0029 (8)	−0.0058 (9)
C7	0.0250 (10)	0.0332 (12)	0.0227 (11)	0.0036 (9)	−0.0061 (9)	−0.0018 (9)
C8	0.0286 (11)	0.0216 (10)	0.0221 (10)	0.0037 (8)	−0.0025 (9)	−0.0014 (8)
C9	0.0282 (11)	0.0299 (12)	0.0293 (11)	0.0035 (9)	−0.0017 (10)	−0.0044 (9)
C10	0.0372 (13)	0.0374 (14)	0.0292 (12)	0.0028 (11)	0.0060 (10)	−0.0088 (10)
C11	0.0334 (12)	0.0390 (14)	0.0221 (11)	−0.0033 (11)	0.0055 (10)	−0.0027 (9)
C12	0.0276 (10)	0.0264 (11)	0.0189 (10)	−0.0076 (8)	0.0006 (8)	−0.0037 (8)
C13	0.0398 (13)	0.0270 (12)	0.0207 (10)	−0.0064 (9)	−0.0014 (9)	0.0010 (9)
C14	0.0389 (13)	0.0255 (11)	0.0299 (11)	−0.0008 (9)	−0.0090 (10)	0.0028 (9)
C15	0.0269 (10)	0.0298 (12)	0.0318 (12)	0.0005 (9)	−0.0013 (9)	−0.0016 (9)
C16	0.0228 (9)	0.0305 (12)	0.0202 (10)	−0.0037 (9)	0.0012 (8)	0.0007 (9)
C17	0.0228 (9)	0.0240 (10)	0.0179 (9)	−0.0048 (9)	−0.0026 (8)	−0.0010 (8)

### Geometric parameters (Å, º)

**Table:**

S1—C1	1.639 (2)	C13—C14	1.379 (3)
N1—N2	1.250 (3)	C14—C15	1.384 (3)
N1—C1	1.460 (3)	C15—C16	1.382 (3)
N2—C2	1.482 (3)	C16—C17	1.393 (3)
N3—C1	1.344 (3)	C4—H4	0.9500
N3—C2	1.475 (3)	C5—H5	0.9500
N3—C3	1.432 (3)	C6—H6	0.9500
C2—C9	1.527 (3)	C7—H7	0.9500
C2—C17	1.521 (3)	C8—H8	0.9500
C3—C4	1.383 (3)	C9—H9A	0.9900
C3—C8	1.385 (3)	C9—H9B	0.9900
C4—C5	1.395 (3)	C10—H10A	0.9900
C5—C6	1.384 (3)	C10—H10B	0.9900
C6—C7	1.380 (3)	C11—H11A	0.9900
C7—C8	1.385 (3)	C11—H11B	0.9900
C9—C10	1.525 (3)	C13—H13	0.9500
C10—C11	1.513 (4)	C14—H14	0.9500
C11—C12	1.509 (3)	C15—H15	0.9500
C12—C13	1.392 (3)	C16—H16	0.9500
C12—C17	1.403 (3)		
			
N2—N1—C1	110.2 (2)	C12—C17—C16	120.0 (2)
N1—N2—C2	112.12 (19)	C3—C4—H4	120.00
C1—N3—C2	110.15 (17)	C5—C4—H4	120.00
C1—N3—C3	125.25 (18)	C4—C5—H5	120.00
C2—N3—C3	123.53 (17)	C6—C5—H5	120.00
S1—C1—N1	121.04 (16)	C5—C6—H6	120.00
S1—C1—N3	132.35 (17)	C7—C6—H6	120.00
N1—C1—N3	106.61 (19)	C6—C7—H7	120.00
N2—C2—N3	100.87 (16)	C8—C7—H7	120.00
N2—C2—C9	108.74 (18)	C3—C8—H8	120.00
N2—C2—C17	106.86 (16)	C7—C8—H8	120.00
N3—C2—C9	111.15 (17)	C2—C9—H9A	109.00
N3—C2—C17	114.04 (17)	C2—C9—H9B	109.00
C9—C2—C17	114.10 (18)	C10—C9—H9A	109.00
N3—C3—C4	120.40 (17)	C10—C9—H9B	109.00
N3—C3—C8	118.97 (19)	H9A—C9—H9B	108.00
C4—C3—C8	120.61 (19)	C9—C10—H10A	110.00
C3—C4—C5	119.16 (19)	C9—C10—H10B	110.00
C4—C5—C6	120.5 (2)	C11—C10—H10A	110.00
C5—C6—C7	119.7 (2)	C11—C10—H10B	110.00
C6—C7—C8	120.5 (2)	H10A—C10—H10B	108.00
C3—C8—C7	119.6 (2)	C10—C11—H11A	109.00
C2—C9—C10	110.90 (19)	C10—C11—H11B	109.00
C9—C10—C11	110.2 (2)	C12—C11—H11A	109.00
C10—C11—C12	113.14 (19)	C12—C11—H11B	109.00
C11—C12—C13	120.0 (2)	H11A—C11—H11B	108.00
C11—C12—C17	122.1 (2)	C12—C13—H13	119.00
C13—C12—C17	117.9 (2)	C14—C13—H13	119.00
C12—C13—C14	122.0 (2)	C13—C14—H14	120.00
C13—C14—C15	119.7 (2)	C15—C14—H14	120.00
C14—C15—C16	119.6 (2)	C14—C15—H15	120.00
C15—C16—C17	120.8 (2)	C16—C15—H15	120.00
C2—C17—C12	120.4 (2)	C15—C16—H16	120.00
C2—C17—C16	119.55 (18)	C17—C16—H16	120.00
			
C1—N1—N2—C2	1.3 (3)	N3—C2—C17—C16	38.4 (3)
N2—N1—C1—S1	176.99 (17)	C9—C2—C17—C12	−16.1 (3)
N2—N1—C1—N3	−2.6 (3)	C9—C2—C17—C16	167.6 (2)
N1—N2—C2—N3	0.3 (2)	N3—C3—C4—C5	178.98 (19)
N1—N2—C2—C9	−116.6 (2)	C8—C3—C4—C5	0.5 (3)
N1—N2—C2—C17	119.8 (2)	N3—C3—C8—C7	−179.93 (18)
C2—N3—C1—S1	−176.77 (17)	C4—C3—C8—C7	−1.4 (3)
C2—N3—C1—N1	2.7 (2)	C3—C4—C5—C6	1.0 (3)
C3—N3—C1—S1	−8.3 (3)	C4—C5—C6—C7	−1.5 (3)
C3—N3—C1—N1	171.17 (19)	C5—C6—C7—C8	0.6 (3)
C1—N3—C2—N2	−2.0 (2)	C6—C7—C8—C3	0.9 (3)
C1—N3—C2—C9	113.2 (2)	C2—C9—C10—C11	−61.8 (3)
C1—N3—C2—C17	−116.1 (2)	C9—C10—C11—C12	49.2 (3)
C3—N3—C2—N2	−170.63 (17)	C10—C11—C12—C13	159.1 (2)
C3—N3—C2—C9	−55.5 (3)	C10—C11—C12—C17	−21.2 (3)
C3—N3—C2—C17	75.2 (2)	C11—C12—C13—C14	179.9 (2)
C1—N3—C3—C4	130.6 (2)	C17—C12—C13—C14	0.1 (4)
C1—N3—C3—C8	−50.9 (3)	C11—C12—C17—C2	4.2 (3)
C2—N3—C3—C4	−62.4 (3)	C11—C12—C17—C16	−179.6 (2)
C2—N3—C3—C8	116.1 (2)	C13—C12—C17—C2	−176.1 (2)
N2—C2—C9—C10	−74.6 (2)	C13—C12—C17—C16	0.2 (3)
N3—C2—C9—C10	175.21 (19)	C12—C13—C14—C15	−0.4 (4)
C17—C2—C9—C10	44.5 (3)	C13—C14—C15—C16	0.4 (4)
N2—C2—C17—C12	104.1 (2)	C14—C15—C16—C17	−0.2 (4)
N2—C2—C17—C16	−72.2 (2)	C15—C16—C17—C2	176.1 (2)
N3—C2—C17—C12	−145.4 (2)	C15—C16—C17—C12	−0.2 (4)

### Hydrogen-bond geometry (Å, º)

Cg3 is the centroid of the benzene ring of the 1,2,3,4-tetrahydronaphthalene group.

**Table:**

*D*—H···*A*	*D*—H	H···*A*	*D*···*A*	*D*—H···*A*
C7—H7···S1^i^	0.95	2.86	3.568 (3)	132
C6—H6···*Cg*3^ii^	0.95	2.84	3.602 (2)	138
C16—H16···*Cg*3^iii^	0.95	2.90	3.676 (3)	139

Symmetry codes: (i) *x*−1, *y*, *z*; (ii) *x*−1/2, −*y*+1/2, −*z*+1; (iii) *x*+1, *y*, *z*.
